# How do survivors after out-of-hospital cardiac arrest perceive their health compared to the norm population? A nationwide registry study from Norway

**DOI:** 10.1016/j.resplu.2023.100549

**Published:** 2024-01-09

**Authors:** Kristin Alm-Kruse, Gunhild M. Gjerset, Ingvild B.M. Tjelmeland, Cecilie B. Isern, Jo Kramer-Johansen, Andrew M. Garratt

**Affiliations:** a Department of Research and Development, Division of Emergencies and Critical Care, Oslo University Hospital, Oslo, Norway; b Faculty of Medicine, Institute of Clinical Medicine, University of Oslo, Oslo, Norway; c National Advisory Unit on Late Effects after Cancer Treatment, Department of Oncology and Department of Clinical Service, Division of Cancer Medicine, Oslo University Hospital, Oslo, Norway; d Division of Prehospital Services, Oslo University Hospital, Oslo, Norway; e Institute for Emergency Medicine, University Hospital Schleswig-Holstein, Kiel, Germany; f Oslo Sports Trauma Research Centre, Department of Sports Medicine, Norwegian School of Sport Sciences, Oslo, Norway; g Division for Health Services, Norwegian Institute of Public Health, Oslo, Norway; h Health Services Research Centre, Akershus University Hospital, Lørenskog, Norway

**Keywords:** Cardiac arrest, Out-of-hospital cardiac arrest, Patient reported outcome, Health, Cardiac arrest registries, PROMs

## Abstract

**Introduction:**

Self-perceived health status data is usually collected using patient-reported outcome measures. Information from the patients’ perspective is one of the important components in planning person-centred care. The study aimed to compare EQ-5D-5L in survivors after out-of-hospital cardiac arrest (OHCA) with data for Norwegian population controls. Secondary aim included comparing characteristics of respondents and non-respondents from the OHCA population.

**Methods:**

In this cross-sectional survey, 714 OHCA survivors received an electronic EQ-5D-5L questionnaire 3–6 months following OHCA. EQ-5D-5L assesses for five dimensions of health (mobility, self-care, usual activities, pain/discomfort, and anxiety/depression) with five-point descriptive scales and overall health on a visual analogue scale from 0 (worst) to 100 (best) (EQ VAS). Results are used to calculate the EQ index ranging from −0.59 (worst) to 1 (best). Patient responses were matched for age and sex with existing data from controls, collected through a postal survey (response rate 26%), and compared with Chi-square tests or t-tests as appropriate.

**Results:**

Of 784 OHCA survivors, 714 received the EQ-5D-5L, and 445 (62%) responded. Respondents had higher rates of shockable first rhythm and better cerebral performance category scores than the non-respondents. OHCA survivors reported poorer health compared to controls as assessed by EQ-5D-5L dimensions, the EQ index (0.76 ± 0.24 vs 0.82 ± 0.18), and EQ VAS (69 ± 21 vs 79 ± 17), except for the pain/discomfort dimension.

**Conclusions:**

Norwegian OHCA survivors reported poorer health than the general population as assessed by the EQ-5D-5L. PROMs use in this population can be used to inform follow-up and health care delivery.

## Introduction

Out-of-hospital cardiac arrest (OHCA) has a high mortality rate, and the survival rate is a common short-term outcome measure.^1^ Parallel to a slight increase in survival and a shift towards patient-centred healthcare,^2^ the 2015 version of the Utstein Resuscitation Registry Templates for OHCA^3^, the current resuscitation guidelines^4^, ^5^ and Core Outcome Set for Cardiac Arrest (COSCA)^6^ recommends assessment of health and quality of life in survivors. Patient-reported outcome measures (PROMs) are largely used for this purpose and assess health from the perspective of OHCA survivors.

Following a literature review and consensus process to propose a core outcome set for cardiac arrest, it was concluded that there is considerable variation in PROMs used to assess health in survivors of OHCA and that none are specific to this population.^7^ It follows that generic PROMs, such as EQ-5D-5L, have had the greatest application in this population.^3^ In spite of the Utstein recommendations for data collection by OHCA registries,^3^ PROMs data are rarely routinely collected.^8^ A few registry-based studies have compared PROMs scores for OHCA survivors and a control population, but with conflicting results.^9^, ^10^, ^11^ Australian OHCA survivors were reported to have favourable health compared to a matched population index.^9^ In contrast, two separate studies from Sweden both reported poorer results across several EQ-5D-domains.^10^, ^11^ Norwegian data collected as part of a prospective clinical trial compare well with the results from Sweden.^12^ The Australian study is the only to present data on an unselected population of OHCA survivors from a cardiac arrest registry. Registry data can provide a unique insight into the self-perceived health of an unselected national population of OHCA survivors. Comparison to a control population is important for the interpretability of the results and particularly in the absence of data pre-cardiac arrest. More and reliable data on health outcomes for OHCA survivors is necessary to inform healthcare personnel, and to tailor guidelines for rehabilitation and follow-up.

This study describes the health of the national register population of Norwegian survivors of OHCA and compares their responses with age- and sex-matched controls from the Norwegian general population (controls). The secondary aim was to compare patient characteristics for OHCA respondents and non-respondents.

## Methods

### Design, setting and participants

The study included the first two years of PROMs data from the Norwegian Cardiac Arrest Registry (NorCAR), 2020–2021. Established in 2002, NorCAR is a national, person-identifiable resuscitation registry.^13^

We included OHCA survivors who were Norwegian citizens with a valid personal identification number, and 18 years or older at the time of cardiac arrest. NorCAR sends the PROM questionnaire to patients that received treatment (chest compressions or defibrillation) started or continued by ambulance personnel, or patients that have circulation at arrival of the ambulance after successful defibrillation by an automated external defibrillator (ROSC by AED).

OHCA survivors were categorised as non-respondents if they did not return the PROM questionnaire. Survivors with more than one OHCA event received one questionnaire per year following the first registered event.

### Procedure and data collection

Survivors with digital access received an electronic invite and secure link to the PROMs through Helsenorge.no, a national platform for communication between healthcare services and patients. A postal version with reply-paid return envelope was sent to those without digital access. Non-respondents received a digital or postal reminder after two weeks.

NorCAR began PROMs data collection in 2021 when the survivors from 2020 were contacted, receiving the questionnaire 3–12 months after cardiac arrest. Questionnaires were then sent quarterly, 3–6 months after cardiac arrest. Questionnaires are sent irrespective of neurological status at discharge from hospital or any level of assisted living.

### The Norwegian population norms for EQ-5D-5L (controls)

Norwegian population norms for EQ-5D-5L, EQ VAS and EQ index, for the adult general population aged 18 years and older, were published in 2021.^14^ To achieve a random group invite, the National Registry of the Norwegian Tax Administration was used for selection based on the estimated sample size per age and sex group. In total, 3200 (26%) responded to the postal survey distributed in 2019.^14^

### Outcome measures

The EuroQol EQ-5D-5L is a widely tested and applied PROM^15^, ^16^ that is recommended in the COSCA statement,^6^ but evidence for measurement properties is lacking in this population.^7^ The instrument assesses five health dimensions: mobility, self-care, usual activities, pain/discomfort, and anxiety/depression.^17^ Respondents rate each dimension on a five-point scale of no problem, slight problems, moderate problems, severe problems, and unable-to-do/extreme problems. The Norwegian version followed EuroQol translation procedures.^18^ The five responses to the health dimensions contribute to a health profile with a 5-digit code (e.g., 12234) reflecting the response categories. The health profile is scored to give a single EQ index using a scoring algorithm from value sets obtained from general population samples. Based on current recommendations for Norway, the UK value set was used^14^ which gives an EQ index score that ranges from −0.59 to 1, where 1 is the best possible health state. In addition to the five dimensions, self-rated health is assessed using a vertical visual analogue scale (EQ VAS), with endpoints labelled “Best imaginable health state” (100) and “Worst imaginable health state” (0).^17^ The EQ-5D-5L has evidence for acceptable measurement properties including reliability and validity, in a range of patient and illness-free populations, also in Norway.^14^, ^15^, ^16^, ^19^

### Statistical analysis

The data were expressed as means ± standard deviation (SD) or median with interquartile range (IQR) and counts with percentages. Reporting follows recommendations based on national applications of the EQ-5D,^20^, ^21^ and we compared groups using Chi-Square tests for the EQ-5D-5L dimensions and independent samples t-tests for the index and EQ VAS scores. The significance level was set at p-value < 0.05. No power calculation was performed because the study was based on available registry data. The controls were randomly matched, in a 1:1 ratio, for age and sex to the OHCA cases.

Respondents and non-respondents to the EQ-5D-5L were compared according to age, sex, cardiac arrest location, bystander resuscitation, ambulance response intervals, shockable first rhythm and neurological state at discharge using the Cerebral Performance Category (CPC – dichotomised to 1–2 good neurological outcome and 3–4 adverse neurological outcome). EQ-5D-5L dimensions, EQ index and EQ VAS scores were compared across seven age groups (18–29, 30–39, 40–49, 50–59, 60–69, 70–79, and 80 and older) and sex.

Case-control matching was undertaken in Stata version 15 (StataCorp). Statistical analysis was performed using IBM SPSS Statistics v28.0 (IBM Corporation).

### Ethics

NorCAR data collection is mandated by law without the need for consent. ^22^ The local data protection officer found the use of collected data to be within the scope of registry regulations and in adherence to the General Data Protection Regulations (case file 11/21096). The steering committee recommended data disclosure for the registry.

### Patient and public involvement

NorCAR steering committee includes a user representative from the patient organisation National Association of Heart and Lung Disease (LHL), who provides a channel for communication to the patient population and the boards of health trusts through a network of fellow user representatives. The representative contributed to the development of this research project.

## Results

**Fig. 1:**
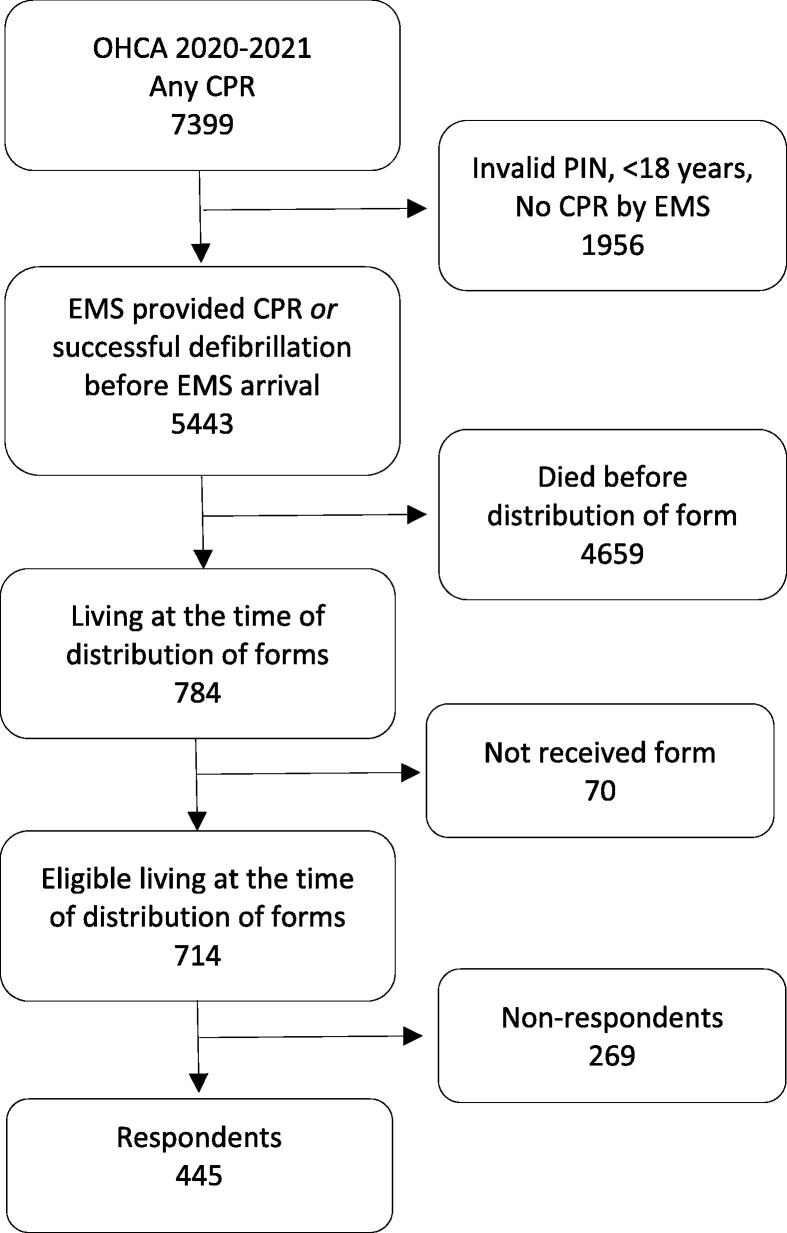
Flowchart of study selection for patients sharing information about health after surviving an out-of-hospital cardiac arrest (OHCA) in Norway 2020–2021. CPR – Cardiopulmonary resuscitation. EMS – Emergency medical services. PIN – Personal identification number. The unit in the diagram is unique patients.

Of the 784 OHCA survivors in Norway for the two years from 1 January 2020 to 31 December 2021, 70 did not receive a questionnaire due to late registration, leaving 714 eligible OHCA survivors, of whom 445 (62%) completed the EQ-5D-5L (Fig. 1). Of the respondents, 89% received the form electronically compared to 71% of the non-respondents (p < 0.001).

Respondents had higher rates of shockable first rhythm and better CPC scores at hospital discharge than the non-respondents. There were no other significant differences (Table 1).

**Table 1:** Characteristics of respondents and non-respondents among out-of-hospital cardiac arrest (OHCA) survivors for the EQ-5D-5L form during 2020 and 2021.

	Respondents n = 445 (%)	Non-respondents n = 269 (%)	Missing or unknownn (%)	p-value
Age, years, median (25-, 75-percentiles)	62 (53, 71)	61 (47, 75)	-	0.88
Males	351 (79)	198 (74)	-	0.11
Location of cardiac arrest			-	0.48
Home	224 (50)	128 (48)		
Other	221 (50)	141 (52)		
Bystander resuscitation*	298 (90)	169 (85)	2 (0)	0.23
Ambulance response interval in minutes, median (IQR)**	7 (6, 10)	7 (5, 11)	6 (1)	0.96
Shockable first rhythm	322 (72)	147 (55)		<0.001
CPC at hospital discharge			118 (17)***	<0.001
1–2 (good neurological outcome)	359(97)	194 (87)		
3–4 (adverse neurological outcome)	13 (4)	28 (13)		
Discharged to home	230 (55)	122 (48)	41 (6)	0.1
Received electronic PROM questionnaire	396 (89)	190 (71)	-	<0.001

CPC – Cerebral performance category score scale.

P-values are from Chi-square tests for categorical data and Mann-Whitney U-test for non-parametrically distributed continuous data.

* *Bystander resuscitation and response interval are reported from the number of OHCA without ambulance-witnessed cardiac arrests, n = 603.

** Ambulance response interval was calculated in minutes between the call answered in the Emergency Medical Communication Centre and when the ambulance stopped at the patient's location.

*** A total of 118patients were missing the CPC score, 73 were respondents, and 45 were non-respondents.

### Outcome measurements

**Fig. 2:**
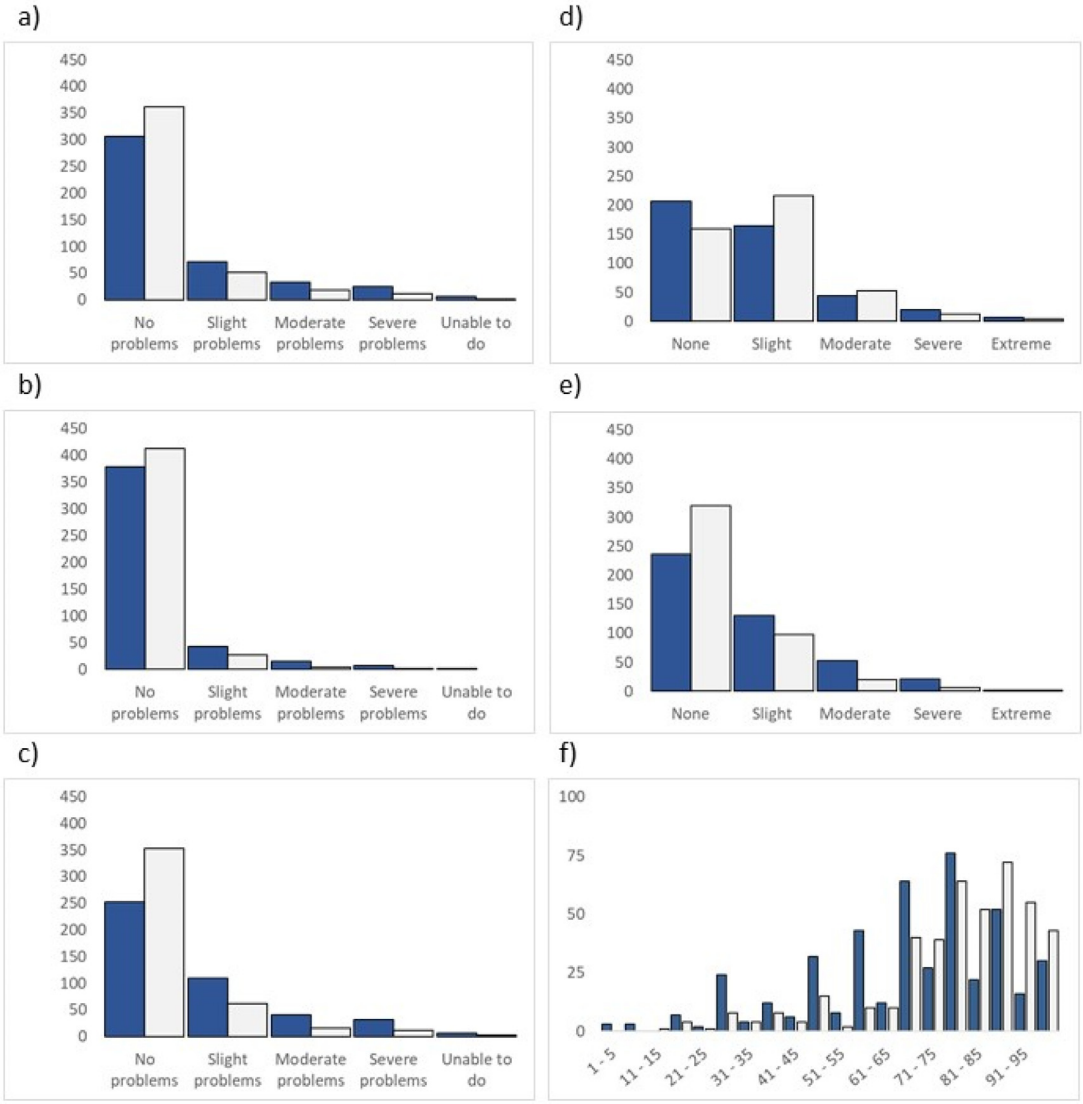
EQ-5D-5L response frequencies for survivors after out-of-hospital cardiac arrest (OHCA) and age- and sex-matched controls from the Norwegian general population. Dimensions are a) mobility, b) self-care, c) usual activities, d) pain/discomfort, and e) anxiety/depression. Panel f) is the EQ VAS for your health today. OHCA survivors are represented with blue columns and the controls with white columns. EQ VAS scores are categorised for purposes of presentation.

**Fig. 3:**
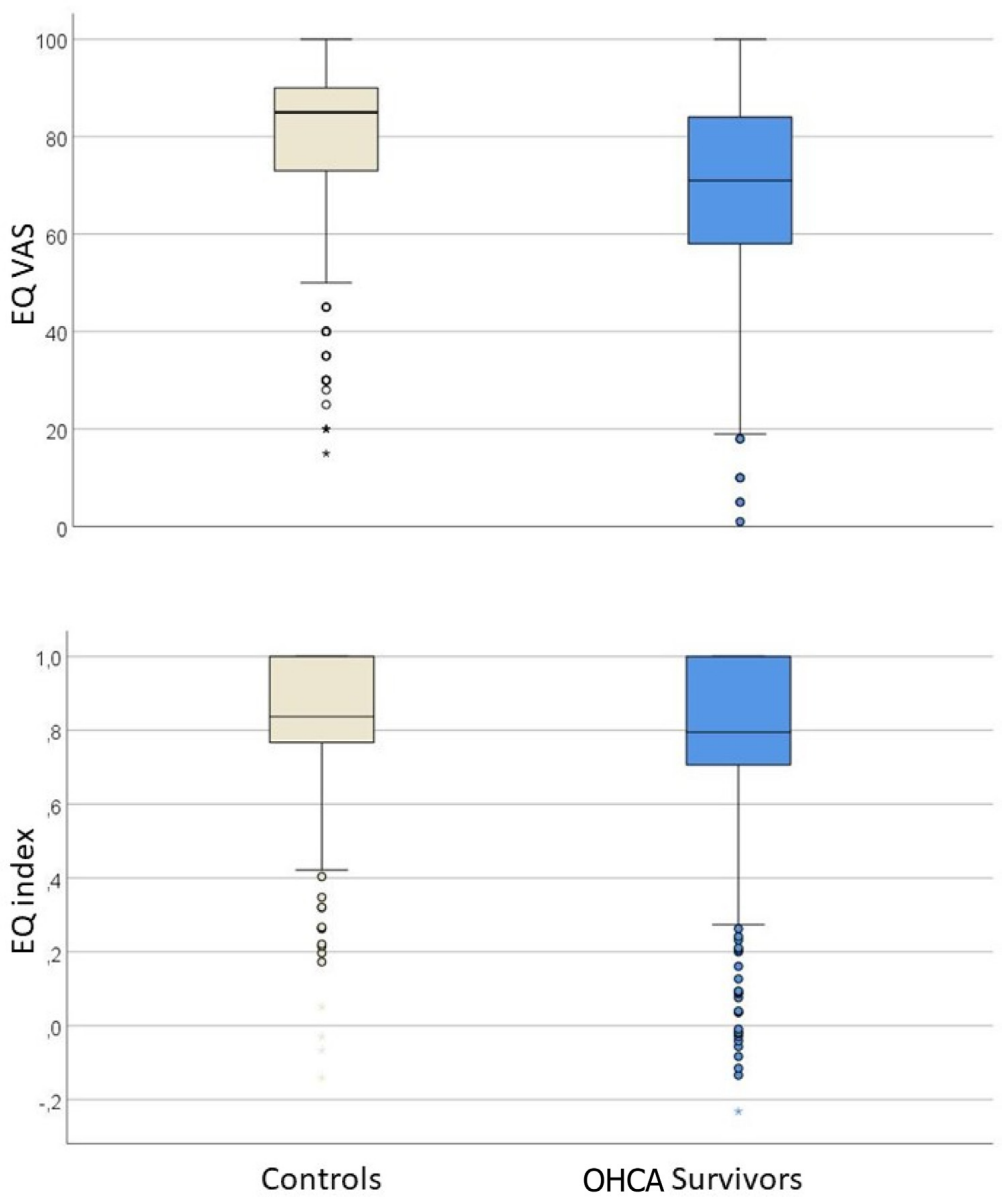
Box and whiskers diagram of the self-reported general health visual-analogue-scale (EQ VAS) and the summary statistic EQ index from the EQ-5D-5L scores for survivors after out-of-hospital cardiac arrest (OHCA) and age- and sex-matched controls from the Norwegian general population. EQ VAS ranges from 0 (worst possible) to 100 (best possible health). EQ index ranges from −0.59 to 1, where 1 is no problems and values below 0 are worse than death. The OHCA survivors have blue, and the controls have white symbols. The box with horizontal line represents 75-, 25- and 50- percentiles, respectively, and whiskers represent 5- and 95-percentiles. Outliers are represented with circles and extreme outliers with stars.

Except for pain/discomfort, OHCA survivors reported poorer health compared to controls across the EQ-5D-5L dimensions, and these differences were significant (p < 0.001) (Table 2 and Fig. 2). The mean EQ-5D-5L index scores for OHCA survivors and controls were 0.76 (SD ± 0.24) and 0.82 (SD ± 0.18), respectively; mean difference 0.054 (95% CI 0.03 – 0.08, p < 0.001). The mean EQ VAS scores for the survivors and controls were 69 (SD ± 21) and 79 (SD ± 17), respectively; mean difference of 10.6 (95% CI 8.1–13.2, p < 0.001) (Table 2 and Fig. 3). There were no significant differences for any EQ-5D-5L scores across age and sex subgroups compared between OHCA survivors and controls. Detailed results are shown in the supplementary figures.

**Table 2:** EQ-5D-5L dimension, EQ VAS and EQ index scores from out-of-hospital cardiac arrest (OHCA) survivors compared with the age- and sex-matched controls from the Norwegian general population.

Dimension/score	Response category	OHCA survivors n = 445 (%)	Controls n = 445 (%)	P-values
Mobility				<0.001
	No problems	306 (69)	362 (81)	
	Slight problems	71 (16)	52 (12)	
	Moderate problems	33 (8)	18 (4)	
	Severe problems	25 (6)	12 (3)	
	Unable to do	7 (2)	1 (0)	
Self-care				<0.001
	No problems	378 (86)	413 (93)	
	Slight problems	42 (10)	26 (6)	
	Moderate problems	14 (3)	4 (1)	
	Severe problems	7 (2)	2 (0)	
	Unable to do	1 (0)	0 (0)	
Usual activities				<0.001
	No problems	253 (57)	353 (79)	
	Slight problems	110 (25)	62 (14)	
	Moderate problems	41 (9)	16 (4)	
	Severe problems	32 (7)	12 (3)	
	Unable to do	6 (1)	2 (0)	
Pain/discomfort				<0.001
	None	207 (47)	160 (36)	
	Slight	164 (37)	217 (49)	
	Moderate	44 (10)	52 (12)	
	Severe	20 (5)	12 (3)	
	Extreme	7 (2)	4 (1)	
Anxiety/depression				<0.001
	None	236 (53)	320 (72)	
	Slight	130 (29)	98 (22)	
	Moderate	53 (12)	20 (5)	
	Severe	21 (5)	6 (1)	
	Extreme	2 (1)	1 (0)	
EQ VAS	Mean (SD)	69 (21)	79 (17)	
	Mean difference (CI)	10.6 (8.1–13.2)		<0.001
EQ index	Mean (SD)	0.76 (0.24)	0.82 (0.18)	
	Mean difference (CI)	0.054 (0.03–0.08)		<0.001

The five dimensions are represented with numbers and percentages. There are three missing responses from OHCA survivors for the five dimensions and two for EQ VAS. From the controls, there are 13 missing responses on EQ VAS. P-values are from Chi-square tests for categorical data. A 95% confidence interval (CI) is given with p-values from an independent samples t-test for EQ VAS and EQ index.

## Discussion

Our study is one of few nationwide studies to report PROMs for survivors following OHCA, that included controls to aid the interpretation of results. Based on responses from 62% of eligible registry respondents, OHCA survivors report poorer health compared to age and sex-matched controls as assessed by four EQ-5D-5L dimensions, EQ index, and EQ VAS scores. The dimension of pain/discomfort was the exception, with OHCA survivors reporting fewer problems than controls.

OHCA outcomes from research studies and registries have mostly focused on survival and functional assessments, but there is increasing interest in how patients themselves perceive outcomes, including aspects of health and quality of life.^6^ The inclusion of the patient perspective is an important addition to outcomes measurement for OHCA because it includes mental, physical, and social aspects of health. PROMs are relevant to clinical and health services research, quality indicators and economic evaluation. In a clinical setting they assess the longer-term impact of OHCA and can inform the selection of care pathways.

Findings from a recent study show that most Norwegian OHCA survivors have considerable pre-cardiac arrest morbidity ^23^ and, compared to general population controls, most likely would have had poorer baseline EQ-5D-5L scores had they been available. Controls facilitate comparisons between different diseases and conditions and increase our understanding of PROM scores, including the use of retrospective measurement. ^24^ However, understanding of OHCA outcomes is hampered by a lack of pre-OHCA PROMs. Future studies comparing, for instance, myocardial infarction patients with and without OHCA could provide valuable additional information on the self-perceived health of patients with cardiovascular comorbidities.

The EQ-5D-5L control data used in this study were collected with the aim of serving as general population reference data to facilitate Norwegian studies in the interpretation of EQ-5D-5L dimension, EQ index, and EQ VAS scores.^14^ Surveys used to collect such data usually have low response rates, potentially introducing selection bias. Where necessary, they are adjusted accordingly including adjustment for age, sex, and education level.^14^ The differences in recruitment procedures and timing of the data collection for the OHCA survivors and the controls could increase the risk of bias, including any COVID-19 related differences in health.

Comparison of PROMs between studies might be affected by several factors and add complexity to the interpretation of the differences. Data collection at different time points following OHCA may limit the comparability because patients may adapt to OHCA-related limitations over time.^25^ Different modes of data collection, including digital, telephone or postal, might also affect responses. Comparable studies have used different versions of EQ-5D, with either three (EQ-5D-3L) or five response levels (EQ-5D-5L), making interpretation more difficult.^16^ The new version with five response levels was chosen for NorCAR based on evidence of improved measurement properties for EQ-5D-5L.^14^, ^16^ However, cardiac arrest-specific PROMs are being developed that have the potential to capture a range of patient's lived experiences and the complex heterogenous nature of recovery and survivorship after cardiac arrest.^26^ Following necessary testing, these should be considered for implementation into quality registers and wider application.

A Swedish study examining health problems among ICD-implanted CA survivors found that CA survivors reported significantly more problems with mobility and usual activities compared to a general population matched for sex and age. ^11^ In addition, our OHCA survivors reported considerably more problems with self-care and anxiety/depression compared to the general population. The Swedish study also found that CA survivors reported significantly higher EQ index scores, and fewer problems with pain/discomfort than the general population. ICD-implanted CA survivors are a selected group and are usually invited to regular hospital follow-ups and could therefore, report better health on these measurements.^11^, ^27^

Compared to controls, fewer of our survivors’ reported problems on the EQ-5D-5L pain/discomfort dimension. It is possible that through contact with health services, OHCA survivors experience better pain management compared to age and sex matched general population controls. This finding is comparable to those from a Swedish register study which found that OHCA survivors reported more problems on all EQ-5D-3L dimensions except pain/discomfort compared to general population controls.^10^

In a Norwegian long-term follow-up after OHCA, health status 5 years after the event was comparable to age- and sex-grouped mean values from the general population, except for lower scores for general health assessed by SF-36. For the EQ-5D dimensions of mobility and self-care, the OHCA survivors reported poorer health compared to the general population.^12^

We found statistically significant differences in EQ-5D-5L index, and EQ VAS scores compared to the controls, but clinical relevance, including minimal important differences (MID), should also be addressed. The mean difference for the index exceeds several suggested estimates for the MID across populations which range from 0.027 to 0.094.^28^, ^29^, ^30^ MIDs for the EQ VAS are less reported,^31^, ^32^ but the mean difference in this study exceeds several of these estimates of 0.5 to 12.0.^33^ Such estimates further aid the interpretation, but application is hindered by variation in terminology and methodology.^34^

High response rates are necessary but not sufficient for external validity. More importantly, there should be no important differences between non-respondents and respondents to the survey. We found significant differences between respondents and non-respondents for proportion with shockable first rhythm and CPC score at discharge. This could signify that fewer responses were obtained from survivors with poorer neurologic outcomes. However, the number of patients discharged with CPC 3 or 4 is low in both groups and only comprises around 5% of the discharged patients. CPC is a blunt neurological outcome measure and may underestimate the level of cognitive impairment hindering self-completion of the questionnaire form. We have no information about who completed the questionnaires, but the included information following the invitation to complete the form, stated that it should be done by the survivors, not proxies. Missing responses complicates the interpretation of the results. The response rate of 62% is similar to that for the Swedish Cardiac Arrest Registry.^35^ Higher response rates were reported for the Victoria Ambulance Registry in Australia, where telephone interviews were used as a routine follow-up 12 months after the cardiac arrest event.^36^

NorCAR uses electronic data collection for EQ-5D-5L with paper forms available if necessary. We choose electronic distribution as the main mode for our registry to minimise cost and work burden on registry staff. PROM scores and measurement properties are generally comparable for electronic and paper administration, but response rates differ across modes of administration.^37^, ^38^ Parallel to a trend in declining response rates to surveys in general,^39^ several studies have assessed the effect of the mode of data collection on response rates^37^, ^40^, ^41^, ^42^ Most report higher response rates with paper rather than electronic surveys.^40^, ^41^, ^42^ These studies were all performed before the COVID-19 pandemic. In the general population, higher response rates have been found for web-based PROMs compared to traditional paper surveys.^37^ This was also found in the current study. The probability of responding to either method has been found to vary according to respondent characteristics, and in the elderly, paper-based administration gave the highest response rate.^37^ The elderly comprise a substantial component of the OHCA population, but our results indicate that functional capacity, rather than age, affects the response rate to the electronic survey. In addition, before we commenced data collection, the COVID-19 pandemic had contributed to Norwegian citizens becoming more electronically active, including booking vaccinations, receipt of test results and arranging doctor appointments. Further research is needed to assess for response bias in this population, including the effect of cognitive impairment on responses to both electronic and traditional modes of data collection.

### Strengths and limitations

The 62% response rate could have contributed to differences between respondents and non-respondents, which in turn may have led to an overestimation of health among OHCA survivors and an underestimation of the differences between OHCA survivors and controls. This possibility for bias could be moderated in the future using methods to increase response rates. In addition, the response rates for OHCA survivors are higher than the controls, adding a layer of complexity to interpreting the results. The controls were not fully representative of the Norwegian population, but matching the respondents and the controls addresses this issue.

EQ-5D-5L is a brief PROM with low respondent burden that gives a general assessment of the cardiac arrest patients' perception of health status, and further testing for measurement properties, including validity, is recommended.^4^ However, the instrument might not include all aspects of health that are important to OHCA survivors. Still, this simple and accessible PROM has allowed us to collect important information from cardiac arrest survivors nationwide, and is recommended for cardiac arrest survivors.^6^ In 2022, a protocol was published, describing the development of a more sensitive and specific PROM measure of cardiac arrest survivorship and self-perceived health. An OHCA specific tool will contribute to future registry research, and in developing follow-up plans for the patients. ^26^ A robust assessment of cardiac arrest survivors’ health will capture important health problems, identify vulnerable subgroups and inform health care delivery. Health status can change over time following OHCA, and longitudinal studies that includes PROMs will enhance understanding of the trajectory.

## Conclusion

Compared to controls, OHCA survivors report poorer health as assessed by four of the five EQ-5D-5L dimensions, EQ VAS scores, and EQ index. The external validity of the PROMs data in NorCAR is somewhat compromised by a higher percentage of non-favourable OHCA event characteristics in non-respondents than in respondents. However, the overall proportion of patients with these characteristics is low, and we consider data to be adequate for the purpose of reporting EQ-5D-5L scores for the overall OHCA population. PROMs can give us information to better understand the experience of the OHCA survivors, which could be useful in the planning of follow-up care for these patients.

## Role of funding source

Funding for this project was received from Laerdal Foundation (grant ID: 2021-0054). The study and the paper were not influenced by this funding.

## CRediT authorship contribution statement

**Kristin Alm-Kruse:** Writing – review & editing, Writing – original draft, Visualization, Project administration, Methodology, Investigation, Formal analysis, Data curation, Conceptualization. **Gunhild M. Gjerset:** Writing – review & editing, Writing – original draft, Visualization, Methodology, Formal analysis, Data curation, Conceptualization. **Ingvild B.M. Tjelmeland:** Writing – review & editing, Writing – original draft, Visualization, Project administration, Methodology, Investigation, Formal analysis, Data curation, Conceptualization. **Cecilie B. Isern:** Writing – review & editing, Writing – original draft, Visualization, Methodology, Formal analysis. **Jo Kramer-Johansen:** Writing – review & editing, Writing – original draft, Visualization, Methodology, Funding acquisition, Formal analysis, Data curation, Conceptualization. **Andrew M. Garratt:** Writing – review & editing, Writing – original draft, Visualization, Methodology, Formal analysis, Data curation, Conceptualization.

## Declaration of competing interest

The authors declare that they have no known competing financial interests or personal relationships that could have appeared to influence the work reported in this paper.

## Appendix A Supplementary material

Supplementary data 1 The EQ-5D-5L dimensions are presented separately for out-of-hospital cardiac arrest (OHCA) survivors and controls in a Norwegian study conducted between 2020 and 2021. The data is categorized by seven age groups and further stratified by sex. The color-coded representation includes dark blue for the mobility dimension, red for self-care, green for usual activities, purple for pain/discomfort, and light blue for the anxiety/depression dimension.
