# Supplementary material for: How do survivors after out-of-hospital cardiac arrest perceive their health compared to the norm population? A nationwide registry study from Norway
Source: Resusc Plus. 2024 Jan 9;17:100549. doi: 10.1016/j.resplu.2023.100549 (PMC10825523; doi:10.1016/j.resplu.2023.100549)
Supplement: Supplementary data 1 — The EQ-5D-5L dimensions are presented separately for out-of-hospital cardiac arrest (OHCA) survivors and controls in a Norwegian study conducted between 2020 and 2021. The data is categorized by seven age groups and further stratified by sex. The color-coded representation includes dark blue for the mobility dimension, red for self-care, green for usual activities, purple for pain/discomfort, and light blue for the anxiety/depression dimension. [file mmc1.docx]

|  | Men | Women |
| --- | --- | --- |
| Age group 18-29 |  |  |
|  |  |  |
| Age group 30-39 |  |  |
|  |  |  |
| Age group 40-49 |  |  |
|  |  |  |
| Age group 50-59 |  |  |
|  |  |  |
| Age group 60-69 |  |  |
|  |  |  |
| Age group 70-79 |  |  |
|  |  |  |
| Age group 80 and above |  |  |
|  |  |  |
